# Annotations

**Published:** 1897-04-17

**Authors:** 


### Annotations.

### Sanitary Pharisees.

English people are not backward in believing, and even in maintaining, that in regard to sanitation England leads the world. Before, however, we thank heaven with too much unction that we are not as other cities—overcrowded, pestilential, and unclean, or even as this poor Bombay which has suffered so severely for her sanitary sins, let us be sure of our facts. Among the evils which have conduced to the present state of affairs in Bombay the greatest possible stress is laid by sanitary experts on the overcrowding of the people. Let us then compare that overcrowding with the condition of certain parts of London. Speaking at the Sanitary Institute a few days ago, Mr. Baldwin Lathom said that the inhabitants of some parts of Bombay are packed together to the extent of 800 individuals per acre; and a correspondent of the *Lancet* writes that in certain districts of Bombay "the density of the population reaches 760 per acre, a figure unequalled anywhere else in the world." If, however, we turn to a description of certain model dwellings in London, recently given in a London police court, we find how far even this "unequalled" figure is exceeded. It was stated that eight blocks of tenements covered about half an acre and were inhabited by 681 persons, or about 1,360 to an acre! We are not here speaking of old rookeries handed down to us from our forefathers, but of "model dwellings" erected in modern times, sanctioned by the authorities, and, presumably, built according to the regulations of Metropolitan Building Acts. We clearly have not much to boast of in regard to the overcrowding which our laws allow. Then as to the result; the death-rate in these dwellings was stated to be 37·8 per 1,000 as compared with 19·0 for the whole of London and 22·9 for the rest of the district in which they were situated, while the death-rate from lung diseases in 1896 was 20·5 within the buildings against 7·3 outside. The public interest of this matter centres in the fact that the plans for the erection of such buildings could be passed by the authorities. The efforts of sanitary committees must be of small avail in keeping down death-rates if so-called improvement committees go on sanctioning the erection of buildings which medical officers condemn almost as soon as they are erected.

### The Fringe of the Profession.

A question has arisen in New York which is exciting considerable interest, viz., whether or not the spectacle-fitters should be licensed! At first sight it seems a small and even a somewhat strained affair, but its analogy to our midwives question is so curious as to make it worth consideration, if even for that reason alone. The medical men claim that the prescribing of glasses is part of their work in the treatment of diseases of the eye, and that the giving of licences to the opticians would be a State recognition of a practice which is contrary to medical law; although they admit that, notwithstanding this law, the practice is and always has been carried on openly and by public consent. The opticians complain that at present anyone can undertake the work of fitting spectacles, that the public cannot tell who is qualified and who is not—just as the midwives complain of their untrained sisters—while a certain section of the medical profession maintains that none of the opticians are qualified at all, and that the spectacle-fitting should all be done by doctors—quite *à la* Rentoul. Some medical men, again, would merely prevent the opticians prescribing glasses for persons under 25 years of age, which curiously recalls to mind the question of what is "normal" and what is "abnormal" in regard to childbirth. It is a pretty little quarrel, and if taken seriously may be looked upon as typical of many other quarrels which may arise at any moment. But are we really to take these things seriously? How about massage, which surely is a mode of medical treatment? If it is wrong for a spectacle-maker to select glasses (and we do not say that it is not very wrong indeed), it must be much more wrong for a medical man to order massage "in the lump," if one may so say, and leave it to the masseur to prescribe the particular proceedings among the many in common use which are applicable to the case. Then about baths and packs and the details of hydropathy; is the keeper of a hydro to be looked on as infringing medical law when he lays down a course of hydropathic treatment? Yet how many doctors are there who could draw up a proper prescription for such a course, or who could off-hand tell even the constituents of a mustard pack or the temperature of the wash down afterwards? Even the cook, we fear, sometimes "usurps" functions which belong to the doctor when she translates into savoury morsels the somewhat crude dietetic prescriptions of the consulting-room. Yet we do not intervene; we all feel that common sense is necessary in such matters, and that it is not dignified to become a laughing-stock.

### Rather Premature.

We do not wish to pose as critics of our medical contemporaries, but is not the following rather premature considering the circumstances? The circumstances were that the *Dilwara*, hired transport, bringing troops from India, arrived at Southampton on April 6th, having had a fatal case of plague on board. Those who were known to have been in immediate contact with the deceased were landed at Moses' Well, disinfection was carried out, and the vessel was allowed to pass through the Suez Canal in quarantine. On reaching Southampton no persons were detained, since all were healthy on arrival—nothing but medical inspection and certain measures of disinfection were necessary, and 1,200 men, women, and children, who, according to the practice of some countries would have been detained in quarantine, were scattered over the land. Now, we have no objection to all this; we have not the slightest doubt that everything was done which was necessary, and that England is just as safe from plague now as she was before the arrival of the *Dilwara*; but surely it is somewhat absurd for a medical paper, which goes to press on April 8th, to speak in the following terms of an experiment like this, which was performed on April 6th! "This case proves anew," says our contemporary, "how easy it is, with proper sanitary administration and healthy conditions on board, to deal with and prevent recurrences of plague, and it also justifies the action laid down by the Local Government Board in dealing with this disease which was formerly subject to quarantine at our ports." As a matter of fact it proves nothing, and we may fairly suggest that such a comment, written, at the latest, on Thursday, on an event which happened on Tuesday, was somewhat premature.

